# Deficits in Facial Emotion Recognition Indicate Behavioral Changes and Impaired Self-Awareness after Moderate to Severe Traumatic Brain Injury

**DOI:** 10.1371/journal.pone.0065581

**Published:** 2013-06-12

**Authors:** Jacoba M. Spikman, Maarten V. Milders, Annemarie C. Visser-Keizer, Herma J. Westerhof-Evers, Meike Herben-Dekker, Joukje van der Naalt

**Affiliations:** 1 Department of Clinical and Developmental Neuropsychology, University of Groningen, Groningen, The Netherlands; 2 Department of Neurology, University of Groningen, University Medical Center Groningen, Groningen, The Netherlands; 3 School of Life Sciences, Heriot-Watt University, Edinburgh, Scotland; 4 Center for Rehabilitation, University Medical Center Groningen, Haren, The Netherlands; 5 Rehabilitation Center Friesland, Beetsterzwaag, The Netherlands; Weill Cornell Medical College, United States of America

## Abstract

Traumatic brain injury (TBI) is a leading cause of disability, specifically among younger adults. Behavioral changes are common after moderate to severe TBI and have adverse consequences for social and vocational functioning. It is hypothesized that deficits in social cognition, including facial affect recognition, might underlie these behavioral changes. Measurement of behavioral deficits is complicated, because the rating scales used rely on subjective judgement, often lack specificity and many patients provide unrealistically positive reports of their functioning due to impaired self-awareness. Accordingly, it is important to find performance based tests that allow objective and early identification of these problems. In the present study 51 moderate to severe TBI patients in the sub-acute and chronic stage were assessed with a test for emotion recognition (FEEST) and a questionnaire for behavioral problems (DEX) with a self and proxy rated version. Patients performed worse on the total score and on the negative emotion subscores of the FEEST than a matched group of 31 healthy controls. Patients also exhibited significantly more behavioral problems on both the DEX self and proxy rated version, but proxy ratings revealed more severe problems. No significant correlation was found between FEEST scores and DEX self ratings. However, impaired emotion recognition in the patients, and in particular of Sadness and Anger, was significantly correlated with behavioral problems as rated by proxies and with impaired self-awareness. This is the first study to find these associations, strengthening the proposed recognition of social signals as a condition for adequate social functioning. Hence, deficits in emotion recognition can be conceived as markers for behavioral problems and lack of insight in TBI patients. This finding is also of clinical importance since, unlike behavioral problems, emotion recognition can be objectively measured early after injury, allowing for early detection and treatment of these problems.

## Introduction

Traumatic brain injury (TBI) constitutes a major global health problem. TBI is estimated to affect approximately 10 million people worldwide per year and is the leading cause of mortality and disability among young adults in Western societies [1], [2]. Many survivors of TBI have residual deficits in cognitive, emotional and behavioral functioning. Behavioral changes are common in patients with moderate to severe TBI [3]–[5] and these are known to have adverse consequences for daily life functioning of patients, negatively affecting social and vocational reintegration and quality of life [6]–[8].

Since these behavioral changes often involve inadequate or inappropriate social-emotional behavior, for example, emotional indifference or hurtful and insulting communication, deficits in social cognition have been put forward by several authors as a possible underlying mechanism [9]–[11]. Social cognition refers to those mental capacities that are assumed necessary to function adequately in the social world and pertains more specifically to the ability to recognize, manipulate and respond to socially relevant information [12]. An important element of social cognition is the ability to recognize facial affect. Facial expressions have important communicatory functions and the ability to read them is considered a prerequisite for understanding other people’s thoughts and feelings and, consequently, for adequate social interaction [10], [13]. Impairments in the ability to recognize facial affect can be demonstrated with neuropsychological, performance-based tests requiring patients to label or match images of facial expressions. In a range of studies, deficits in emotion recognition were found in patients with a moderate to severe TBI [9], [11], [14]–[19], irrespective of age, stage of recovery or type of stimuli used. Ietswaart and colleagues [20] concluded that these deficits tend to be rather stable over time, as they found little spontaneous recovery of emotion perception at one year post injury. In a previous study in patients with moderate to severe TBI [19] we found that, out of a range of social cognition and general cognition measures, emotion recognition was most sensitive to the effects of TBI, and was the only measure that was related to the presence of focal prefrontal damage. This latter finding converges with studies that assign an important role to prefrontal areas in emotion recognition [21]–[23] as well as with the fact that prefrontal areas are known to be specifically vulnerable to TBI [24]–[28]. Moreover, the presence of social behavioral problems is related to damage to inferior and medial prefrontal areas [5], [29]. Consequently, since emotion recognition is considered important for intact social behavior and is frequently impaired in TBI, impairments in emotion recognition might be a significant predictor of behavioral deficits.

To date, few studies have specifically addressed this question. Milders and colleagues [9], [30] found no significant association between facial affect recognition and behavioral deficits, as indicated by proxy reports of patients’ emotional and social behavior or between patients’ facial affect recognition and their level of social integration, either shortly after TBI or in the chronic stage. However, both Knox and Douglas [31] and Struchen and colleagues [32] found a significant relationship between expression recognition and a measure for social integration that was derived from a scale aimed to assess societal and daily life functioning in several domains, the Craig Handicap Assessment and Reporting Technique (CHART, [33]). This social integration subscale can be considered an indirect measure of behavioral deficits, as successful social integration suggests the absence of disturbed behavior. However, although the CHART is administered by a professional and its items focus on observable criteria, the scores are based on self-reported functioning of the patient. Hence, to date no association has been found between impaired facial affect recognition and behavioral deficits as rated by significant others.

An important question in this context is whether a more accurate impression of the patient’s behavior is given by self or proxy reports. Although not immune to bias, relatives’ ratings of patients’ behavior are generally considered as more objective and presenting a more accurate report of patients’ daily functioning than self reports. Self-report measures are very vulnerable to confounding variables, such as lack of insight in the patients. Several studies have demonstrated lack of insight to be a common consequence of moderate to severe TBI, causing patients to provide unrealistically positive reports of their own functioning [34]–[36]. Direct comparisons of self ratings and proxy ratings of behavioral changes following TBI using the Dysexecutive Questionnaire (DEX, [37]), a well-established scale for measuring behavioral changes after brain injury, showed that patients indicated significantly fewer difficulties than their relatives, which was interpreted as an indication of impaired self-awareness in the patients [38], [39]. However, Spikman and colleagues [36] found that impaired self-awareness specifically affected moderate to severe TBI patients with evidenced frontal lesions, whereas patients without frontal lesions demonstrated a more adequate perception of their actual level of functioning.

Another complicating factor when investigating associations between emotion recognition and behavioral ratings might be that the concept of behavioral deficits is too broad and also incorporates post traumatic changes that are not due to social cognition deficits. For instance, inadequate social behavior might also pertain to passivity and lack of interest resulting from increased fatigability or to disorganized behavior related to executive function deficits. As a result, the proportion of variability in post-injury behavior explained by emotion recognition deficits might be small if the measures of post-injury behavior cover a broad range of behaviors. This applies specifically to the DEX, which aims to measure the behavioral changes collectively known as dysexecutive syndrome. This syndrome closely resembles what was once called the ‘frontal lobe syndrome’, characterized by changes in emotion, psychosocial behavior and executive function [37]. Although the DEX originally had no subscales, a factor analysis on the norm sample revealed three different factors that were labeled Behavior, Cognition and Emotion [37]. More recent studies applied factor analyses to fractionate the DEX using the self rating version in samples of neurological patients [40], [41]. Both studies identified one scale that could be conceived as representing changes in psychosocial behavior, consisting of items sensitive to inappropriate and socially inadequate behavior, such as disregard for how others feel about the patient’s behavior. Recently, Simblett and Bateman [42] employed a Rasch analysis to unravel the structure of the DEX self rating version in a sample of 363 patients with acquired brain injury. The authors proposed a division into three subscales, following Stuss’ [43] proposal of different functional dimensions within the prefrontal cortex, called Metacognitive processes, Executive Cognitive functions and Behavioral Emotional Self-regulatory functions, respectively. The last function would be particularly relevant for psychosocial behavior.

The aim of the present study was to investigate whether facial emotion recognition, as measured with a performance-based test, might be a predictor of behavioral deficits in TBI patients, as measured by proxy ratings. In particular we wanted to explore whether emotion recognition, as a crucial aspect of social cognition, would predict those subscales that aim to measure psychosocial behavior. Finally, a question that has not been investigated before is whether deficits in affect recognition were associated with impaired self-awareness in patients with TBI.

## Methods

### Ethics Statement

This study was performed in compliance with the ethical regulations of our institution (UMCG). For 25 of the patients test results were collected as a part of regular clinical follow-up. For these patients and for the healthy controls no medical ethical approval was required (Wet Medisch-Wetenschappelijk Onderzoek met Mensen (Law on Medical-Scientific Research in Humans) Article 1, part 1b and 2). For 26 of the patients the data were collected as part of the inclusion procedure for a study on behavioral sequelae after TBI, which was approved by the medical ethical committee of the University Medical Center Groningen, the Netherlands. All participants gave informed written consent prior to study inclusion for their information to be used in the hospital database and used for research, and were treated in accordance with the declaration of Helsinki.

### Participants

The patient group consisted of 51 moderate to severe TBI patients (defined by a Post Traumatic Amnesia (PTA) duration of 1 day or more or a Glasgow Coma Scale (GCS) score lower than 13), the majority of whom had previously been admitted to the Neurology department at the University Medical Center (UMCG) in Groningen, the Netherlands, a level-one trauma center. At the time of testing, all patients were outpatients who were seen in the sub-acute or chronic stage for clinical follow-up by the trauma neurologist or the rehabilitation physician. Clinicians referred patients as part of routine follow-up for neuropsychological testing to assess possible behavioral problems. For all patients PTA data were available; the mean PTA duration was 32.7 days (SD 34.5), with a range from 1 to 150 days. For 40 patients GCS scores were available, lowest GCS ranging from 3 to 14, with a mean of 8.4 (SD 3.7). The mean Time since Injury (TSI) of this group was 75 months (SD 102) with a broad range varying from 5 months to 34 years. Exclusion criteria for this study were: more than one TBI, neurological conditions other than TBI (e.g. strokes, tumor, seizures, neurodegenerative disorders), psychiatric conditions (e.g. major depression, bipolar disorder, autism, schizophrenia, other conditions requiring admission to a psychiatric ward) and substance abuse pre- or post injury. Fifty-one TBI patients (34 males, 17 females) were included with a mean age of 37.5 years (SD 14.9, range 17–66) and a mean educational level of 5.0 (SD 1.0, range 2–7) (7-point scale ranging from 1 (primary school education only) to 7 (university education)).

The proxies of the patients were partners (n = 31), parents or other family members (n = 13), friends or acquaintances (n = 7), who were contacted by the neuropsychologist and who agreed to fill out the DEX proxy version.

Thirty-three healthy controls (17 males and 16 females) with a mean age of 37.9 (SD 13.2, range 20–60) and a mean educational level of 5.3 (SD 1.2, range 3–7) were recruited by means of an advertisement in a local newspaper. Their proxies were asked to participate by the neuropsychologist. Exclusion criteria were the same as for patients, with brain injury as an additional exclusion criterion. Chi-Square and t-tests showed that the patient and control group were matched for: sex (Χ^2^ = 1.93, p = 0.17), age (t = −0.14, p = 0.89) and educational level (t = −1.29, p = 0.20).

### Measures

#### Emotion perception

The FEEST (Facial Expressions of Emotion- Stimuli and Tests, [44]) is a test for recognition of emotional expressions on faces. It consists of two subtests, the Ekman 60 Faces test, which we used here, and the Emotion Hexagon test. In the Ekman 60 Faces test sixty faces are shown and the expressions depicted are the primary emotions Fear, Disgust, Anger, Happiness, Sadness or Surprise (ten of each). Stimuli are presented for 5 seconds, after which the subject has to choose which emotion label best describes the emotion shown. The total score ranges from 0–60, the separate emotion scores range from 0–10. The authors of the FEEST reported significant split-half reliabilities for the total score and for all emotion scores except Happiness, which did not correlate significantly across the two sets of pictures because scores were at ceiling level. Validity was also satisfactory; recognition rates of the norm group were compared to those of an earlier group of healthy controls, resulting in a high correlation between the two sets of 0.81 [44]. The Ekman 60 Faces test has proven to be sensitive to pathology in other patient groups, for instance Huntington patients [45] and patients with Frontotemporal Dementia [46].

#### Behavioral deficits

The Dysexecutive Questionnaire (DEX, [37]) is a 20-item questionnaire measuring a broad spectrum of behavioral problems that are considered part of the dysexecutive syndrome [38]. The DEX has a self rating and proxy rating version. Higher scores represent more severe problems. Both total scores (DEX-self and DEX-proxy) were used as well as a difference score (DEX-dif = DEX-self minus DEX proxy) as an indication of self-awareness, with a negative difference score indicating an impairment in self-awareness.

Burgess et al. [38] found that both the DEX-proxy and DEX-dif scores were ecologically valid because both showed significant correlations with executive function tests in a large group of neurological patients. Chaytor and Schmitter-Edgecombe [40] reported adequate internal consistency and reliability for the DEX-proxy (α = 0.90).

The individual DEX items are displayed in Table 1.

**Table 1:** Individual DEX items.

1. problems with abstract thinking2. impulsivity,acting without thinking3. confabulation4. planning problems5. euphoria,excitability6. temporal sequencing problems7. lack of insight and social awareness8. apathy and lack of drive9. disinhibition, inappropriate behavior10. variable motivation	11. shallow affect12. losing temper, aggression13. lack of concern14. perseveration15. restlessness16. inability to inhibit responses17. knowing-doing dissociation18. distractibility19. loss of decision making ability20. unconcern for social rules

DEX subscale scores were derived from recent re-analyses of the DEX. In the current study we applied these subscales to the DEX proxy ratings. Chaytor and Schmitter-Edgecombe [40] defined a social-emotional behavioral scale, called “Social inhibition”, on the basis of a factor analysis on the DEX-self in a group of 46 adults with brain injury. Bodenburg and Dopslaff [41] factor analyzed the DEX-self ratings in a larger sample of 191 brain injury patients and found four scales, one of which was interpreted as a Social Convention scale (DEX-SC). This scale overlapped to a large extent with the scale of Chaytor et al. and consisted of items 9, 12, 13 and 20. Bodenburg and Dopslaff’s [41] analysis showed that item 11 (shallowing of affective responses) was not sufficiently discriminating. Therefore, in the present study we used the Bodenburg and Dopslaff DEX-SC scale, measuring awareness of social conventions and the ability to incorporate social interaction in one’s own behavior.

Simblett and Bateman [42] also discarded item 11 on the basis of their Rasch analysis. They defined the following three scales, which we used in addition to the DEX-SC scale:

Behavioral-emotional Selfregulation (DEX-BESR: items 3, 7, 8, 10, 13, 14, 15 and 17), Executive Cognition (DEX-EC: items 1, 4, 6, 18) and Metacognition (DEX-MC items 2, 5, 12, 16, 20).

### Statistical Analyses

Tests of normality of data indicated that the FEEST subscores were not normally distributed. Therefore, for these scores we used non-parametric tests to test for differences between the performance of the TBI patients and the healthy controls. For the FEEST total score and DEX scores *t*-tests were used for between group comparison. One-tailed p values were chosen, as the patient group was expected to perform more poorly, based on previous literature. Effect sizes (Cohen’s d) were calculated for all comparisons between the groups. Pearson correlations were calculated to determine the relationships between the FEEST scores, the DEX-proxy, DEX-self and DEX-dif scores, and the four DEX subscales. For all analyses, alpha levels were adjusted for multiple comparisons using the Bonferroni Holm correction [47]. This is a sequentially rejective version of the simple Bonferroni correction for multiple comparisons, with varying alpha levels, depending on the number of comparisons.

## Results

Table 2 shows the means and SD’s of the FEEST subscores, the FEEST total score and the DEX-self, DEX-proxy and DEX-dif scores for the two groups, together with the results of the between-group comparisons (Mann-Whitney U and t-tests) and the effect sizes. The patients performed significantly poorer than healthy controls on the total FEEST score as well as on the negative emotions Anger, Fear, Sadness and Disgust. The patients reported on average significantly more behavioral problems than the healthy controls on the DEX. Proxy ratings also indicated that patients’ behavioral functioning was significantly poorer than that of the healthy controls. An ANOVA comparing the DEX proxy ratings revealed no significant differences between the three proxy groups i.e. partners, parents/family members and friends or acquaintances (F = 0.36, p = 0.70).The difference on the DEX-dif between the patients and controls was significant. In the patient group DEX-proxy scores were on average higher (suggesting more severe problems) than the DEX-self score. On the other hand, within the control group, proxy ratings tended to be lower than self ratings. The effect sizes for the significant differences ranged from.60 to 1.14, which can be classified as medium to large according to Cohen [48]. There was no significant correlation between time since injury (TSI) and the test measures; FEEST (r = −0,01, p = 0.99), DEX-proxy (r = 0,21, p = 0.14) or DEX-self (r = 0.21, p = 0.15).

**Table 2:** Comparison of FEEST and DEX scores of TBI patients and healthy controls.

Measure	TBI patients (n = 51)	Healthy controls (n = 33)	Z	p	Sign.	d
	M (SD)	M (SD)				
**FEEST Anger**	7.25 (1.9)	8.76 (1.1)	−3.8	.000	*	0.84
**FEEST Disgust**	6.37 (2.9)	7.94 (1.7)	−2.3	.011	*	0.60
**FEEST Fear**	5.04 (2.5)	6.73 (2.2)	−2.9	.002	*	0.68
**FEEST Happiness**	9.78 (0.6)	9.97 (0.2)	−1.8	.033	n.s.	0.40
**FEEST Sadness**	5.86 (2.3)	8.03 (1.7)	−4.5	.000	*	0.90
**FEEST Surprise**	8.78 (1.3)	8.94 (1.0)	−0.3	.365	n.s.	0.13
			**T**			
**FEEST Totalscore**	42.98 (7.2)	50.36 (3.6)	−6.2	.000	*	1.05
**DEX-Self**	27.98 (13.1)	20.61 (7.8)	3.2	.001	*	0.62
**DEX-Proxy**	33.02 (12.2)	17.61 (9.4)	6.2	.000	*	1.14
**DEX-Dif**	−5.04 (15.1)	3.00 (8.9)	−3.1	.002	*	0.60

* Significant p value<Bonferroni Holm corrected alpha.

FEEST:Facial Expressions of Emotions-Stimuli and Test. DEX: Dysexecutive Questionnaire. DEX-self: Self rating version. DEX-proxy: Proxy rating version. DEX-dif: Difference score.

Pearson correlation coefficients between the FEEST sub- and total scores and the DEX-self, DEX-proxy and DEX-dif scores for the TBI patients are shown in Table 3. No significant correlations were found between any of the FEEST variables and the DEX-self rating scores. However, there were significant correlations between the FEEST total score, FEEST Sadness score and DEX-proxy ratings; a lower score on these FEEST scores was associated with more problems on the DEX-proxy scale. Furthermore, FEEST total score and the Anger and Sadness scores were significantly correlated with DEX-dif scores. Thus, lower FEEST scores were associated with poorer self-awareness in the patients, as indicated by a larger negative difference between self ratings and proxy ratings.

**Table 3:** Pearson correlation coefficients for the FEEST and the DEX scores in TBI patients.

FEEST	DEX-Self	DEX-Proxy	DEX-Dif
**Anger**	0.27	−0.19	**0.39** *
**Disgust**	0.09	−0.21	0.24
**Fear**	0.05	−0.36	0.33
**Happiness**	−0.03	0.11	−0.11
**Sadness**	0.09	**−0.47** *	**0.46** *
**Surprise**	−0.09	0.02	−0.09
**Totalscore**	0.14	**−0.38** *	**0.44** *

* Significant p value<Bonferroni Holm corrected alpha.

FEEST:Facial Expressions of Emotions-Stimuli and Test. DEX: Dysexecutive Questionnaire. DEX-self: Self rating version. DEX-proxy: Proxy rating version. DEX-dif: Difference score.

Table 4 shows Pearson correlation coefficients between the FEEST sub- and total scores and the four different DEX subscales that were calculated from the patient group proxy scores. The FEEST total score and the FEEST Sadness score showed significant correlations with the DEX-SC and the DEX-MC scale. In addition, the FEEST Sadness score correlated significantly with DEX-BESR. Finally, the FEEST Fear score showed a significant correlation with the DEX-EC scale. All correlations were negative, indicating that lower scores on the FEEST variables (poorer performance) corresponded with higher scores on the DEX subscales (more problems).

**Table 4:** Pearson correlation coefficients for the FEEST with DEX subscales in TBI patients.

FEEST	DEX-SC	DEX-BESR	DEX-MC	DEX-EC
**Anger**	−0.22	−0.21	−0.15	−0.15
**Disgust**	−0.27	−0.09	−0.33	−0.05
**Fear**	−0.24	−0.30	−0.28	**−0.38** *
**Happiness**	0.01	0.10	0.08	0.17
**Sadness**	**−0.43** *	**−0.40** *	**−0.42** *	−0.31
**Surprise**	−0.01	0.07	−0.01	−0.07
**Totalscore**	**−0.38** *	−0.30	**−0.38** *	−0.29

* Significant p value<Bonferroni Holm corrected alpha.

FEEST:Facial Expressions of Emotions-Stimuli and Test. DEX: Dysexecutive Questionnaire. BESR: Behavioral-emotional Selfregulation Scale. SC: Social Convention Scale. MC: Metacognition Scale. EC: Executive Cognition scale.

## Discussion

To our knowledge, this is the first study that found a significant relationship between deficits in affect recognition after moderate to severe TBI, objectified with a performance based test, and behavioral changes reported by significant others. Our finding substantiates the assumption that deficits in aspects of social cognition may underlie, at least in part, behavioral deficits after brain injury. Moreover, this is the first study to find an association between deficits in emotion recognition and impaired self-awareness of limitations in daily functioning in TBI patients. Hence, poor affect recognition might be an indication of limited insight following brain injury that is known to impede successfull social and vocational reintegration [49].

In line with previous findings we found that moderate to severe TBI patients were significantly impaired in emotion recognition, when compared to a matched group of healthy controls. Patients were impaired on the overall score on the FEEST, as well as on the individual emotion scores, except Surprise and Happiness. Previous studies have suggested that TBI patients are specifically impaired in the recognition of negative emotions, and Fear in particular [14], [50]. However, Ietswaart and colleagues [20] concluded that there was no selective deficit in the recognition of negative emotions, since healthy controls also had more difficulty recognizing the same emotions. Ietswaart and colleagues [20] found both for patients and controls that expressions of Fear were recognized worst, followed by Anger or Disgust, followed by Sadness, Surprise and Happiness. This is in line with findings from previous studies in TBI patients [51] or in patients with brain damage due to various etiologies [52] which found recognition of facial expressions of Fear to be most severely impaired. Furthermore, in healthy normal subjects from various cultures facial expressions of Fear are typically recognised more poorly than any other expression [53], [54]. We also found that Fear was recognized most poorly by both patients and healthy controls, but unlike Ietswaart et al. [20] the second most poorly recognized emotion in our patient group was Sadness. In fact, in terms of the discrepancies between the means of patients and healthy controls and the effect sizes, recognition of Sadness was more severely affected than any of the other emotions. Even more remarkable, recognition of Sadness in the patient group had the strongest correlation with behavioural deficits, as rated by proxies.

Furthermore, we found that the scores of the patient group on both the DEX-self rating version and the DEX-proxy rating version were significantly higher than those of the healthy controls, indicating the presence of behavioral problems in the TBI patients. Proxy ratings of the patients tended to be higher (i.e. indicating more problems) than their self ratings whereas the reverse was true for the healthy controls. The discrepancy was expressed in the DEX-dif score; the mean negative score indicated that patients reported fewer problems than their relatives, which was interpreted as a sign of impaired self-awareness (ISA) or lack of insight. This finding is in line with a range of studies reporting ISA after moderate to severe TBI [35], [36]. What the current study showed was that the extent of the self-awareness deficit in the patients was associated with the severity of emotion recognition deficits. Larger discrepancies between self and proxy ratings correlated with poorer emotion recognition. Again the recognition of Sadness had the strongest correlation with impaired self-awareness. The fact that we found no significant correlations between emotion recognition and the DEX-self rated version is in line with the assumption that self-ratings reflect the actual behavioral status of the patient less accurately than proxy ratings.

However, a previous study by Milders et al. [30] in patients with TBI found no association between impaired emotion recognition and DEX proxy ratings. But in this study the TBI patients were assessed at one year post injury whereas in our current study time since injury was on average more than six years. Bennett and colleagues [55] found proxy ratings on the DEX to be more useful in identifying dysexecutive deficits of subacute TBI patients than self ratings, but ratings by professionals were even more accurate. The authors explained this finding by argueing that significant others need time to adopt an adequate perspective on the present functioning of the patient. Hence, one year post-injury might still be too short to accomplish such adaptation and when time since injury increases proxy ratings might become more accurate. This might explain why in our study the proxy rated DEX score was significantly related to measures of affect recognition in contrast to the Milders et al. study [30].

As pointed out in the introduction, the DEX is a broad measure of behavioral symptoms, designed to cover the full range of symptoms of the dysexecutive (or ‘frontal lobe’) syndrome, that is, changes in emotion, personality, motivation, behavior, executive function and cognition [38]. We expected affect recognition, as an element of social cognition, to be specifically related to subscales that are assumed to measure aspects of psychosocial behavior. To this end DEX- proxy ratings subscales were construced based on results of recent factor analyses [41] or Rasch analyses [42]. Indeed, the total FEEST score as well as the Sadness score showed significant correlations with the Social Convention and Metacognition scales, both assumed to represent the ability to show appropriate social behavior and to keep to social conventions. Nevertheless, these correlations were comparable in size to the correlations with the total DEX-proxy score, indicating that there was no major difference between predicting the total DEX or its psychosocial subscales. The Sadness score, but not the total FEEST score, showed a significant correlation with the DEX-BESR score. A significant correlation with emotion recognition was expected because, according to Simblett and Bateman [42], the DEX-BESR scale can be interpreted as measuring functions that are involved in emotional and reward processing, necessary for appropriate adaptive responding to others, and thus for adequate psychosocial behavior. The Sadness subtest of the FEEST was the only subtest that showed significant correlations with all three behavioral DEX scales, but not with the DEX Executive Cognition subscale. There was one emotion score that correlated with the Executive Cognition subscale and not with any other scale, and that was recognition of Fear. Although this DEX-EC scale measures executive functions (planning, regulation, focussing and switching) that can be considered as non-social cognition, affect recognition had some influence on this scale. A possible explanation might be that deficits in fear perception are related to lower levels of fear experience, which might lead to more impulsive and risk taking behavior. In turn, impulsive and risk taking behavior could interfere with a thoughtful, planned and controlled task approach. Risk taking behavior, impaired fear perception and lower levels of fear experience have been found in subjects with psychopathic traits [56], [57].

We conclude that correlations between emotion recognition and the social-behavioral subscales were not substantially different from the correlations with the total proxy score. A possible explanation might be that we constructed the subscales for the DEX proxy ratings on the basis of analyses performed on DEX patient self ratings. Using Rasch analysis, Chan and Bode [58] found that, even when average scores of TBI patients and proxies on the DEX were comparable, there was differential item functioning resulting in only a moderate relationship between the patient and proxy ratings. This suggests that scales derived from patient data might not fit the proxy data very well and that more optimal scales should be derived from factor or Rasch analyses of large proxy samples. An additional point regarding the measurement of these problems is that, although the social-behavioral subscales are assumed to measure impaired psychosocial behavior, to date the validity of these subscales has not been well established. However, the validity of the total DEX score as measure of behavioral problems following TBI has been demonstrated by strong correlations between DEX total scores and other measures of behavioral difficulties, like the Neuropsychology Behavior and Affect Profile (NBAP) and the Katz Adjustment Scale Revised (KAS-R) [30].

There are other limitations to our study.The patients were not all recruited through random or consecutive selection as part of the group had been referred for assessment of possible behavioral problems, although these were not further specified. However, one could argue that this selection narrowed the range of possible outcomes and thus decreased variability among patients, making it more difficult to find significant correlations. In addition, the broad variation in time since injury might be a limitation as recovery stage might influence test results and ratings. However, Ietswaart and colleagues [20] found little recovery of affect recognition over time, and hence, comparison of early and late measures could be justified. Moreover, we found no evidence for significant relationships between time since injury and the FEEST and DEX scores. Another limitation is that we could not guarantee that all participating patients were free of pre-history personality problems that might have influenced relevant measures, such as a lack of empathy or inability to understand other people’s thoughts and feelings, even though we excluded patients with a history of psychiatric problems. Furthermore, although we found affect recognition deficits as expected using the FEEST, this test can be criticized for being not very ecologically valid as its stimuli from the Ekman and Friesen set are black and white photographs that are visually outdated, presenting only basic emotional expressions and subjects have to respond by means of a forced choice paradigm. However, the Ekman and Friesen set is still widely used in neuropsychological studies and a recent meta analysis of emotion recognition in patients with TBI by Babbage et al. [11] showed that effect sizes in studies that used the FEEST or the Ekman and Friesen set were not systematically different from the effect sizes in studies that used other face sets. Therefore, based on these results there is no reason to assume that the FEEST is more or less difficult for patients. Nevertheless, it would be interesting to repeat this study using an ecologically more valid measure of emotion recognition,such as the TASIT [59]. A final point concerns the relationship between emotion recognition and behavioral changes, which is not necessarily a causal one. Other factors might influence this relationship, for instance impairments in non-social cognitive functions (speed of information processing, attention, executive functioning), which are frequently found in moderate to severe TBI patients. However, we do not consider this possibility very likely as we found in a previous study that deficits in emotion recognition were unrelated to deficits in non-social cognitive functions, even though patients were impaired in both [19].

In conclusion, the ability to recognize facial expressions of emotions was impaired in moderate to severe TBI patients and was significantly associated with a broad range of behavioral problems as rated by a significant other of the patient. This strengthens the proposal that recognition of social signals is a condition for adequate social functioning. In line with many previous studies, we found only negative emotions to be affected, but different from other studies, we found that the ability to recognize Sadness was most severely affected in the patients. Facial expression recognition was correlated with the proxy rating, as well as with a negative discrepancy between self and proxy rating, indicating lack of insight in the patient, but was unrelated to the patients’ self reported behavior post-injury. Particularly strong correlations between DEX proxy rating and patients’ ability to recognize Sadness were found. Thus, when patients are less able to recognize this emotion on other people’s faces, their proxies rate them as more behaviorally disturbed. In addition, the overall ability to recognize emotions as well as the specific abilities to recognize Sadness and Anger were significantly correlated with the DEX-dif score, indicating that when patients were less able to recognize these emotions the contrast between their proxies’ ratings of their behavior changes and their own rating was larger. This is an important finding and we are not aware of studies that have demonstrated this before. This finding suggests that the recognition of Sadness and Anger in others is important for the ability to regulate one’s own social behavior. Patients who are impaired in this ability show more behavioral problems and have less insight. This conclusion is in line with the crucial communicatory functions of these facial expressions as stressed by Blair [13]. He suggests that both sad and angry facial expressions are powerful signals to others that their current behavior has to stop or at least not to be exerted again in the future. It is easily conceivable that when these facial signals are neglected, social interactions and consequently, the relationship with the significant other will be negatively affected. In addition, it is likely that patients who do not recognize Sadness show less empathy with others which might be judged by partners and relatives as a serious behavioral problem. Hence, in particular recognition of Sadness, but also of Anger, might be an important starting point for treatment aimed to improve social behavior. Moreover, since the measurement of both behavioral changes as well as impaired self-awareness in an early stage after TBI may be difficult and less reliable, deficits in affect recognition, in particular in the recognition of Sadness, may be considered a useful marker of those problems that interfere with successful social reintegration.
